# Hierarchical Au–Pt Nanostructured Film-Enabled Electrochemical Sensor for Highly-Sensitive Determination of Salvianolic Acid B

**DOI:** 10.3390/bios16090508

**Published:** 2026-09-10

**Authors:** Yujiao Hou, Fang Lin, Xiang Gao, Jianhua Yang, Longfei Sun, Weijun Kong

**Affiliations:** 1 College of Traditional Chinese Medicine, Xinjiang Medical University, Urumqi 830017, China; houyuj117@163.com; 2 Institute of Traditional Chinese Medicine, Xinjiang Medical University, Urumqi 830017, China; 3 College of Pharmacy, Xinjiang Medical University, Urumqi 830017, China; 17351013705@163.com (F.L.);; 4 Department of Pharmacy, The First Affiliated Hospital of Xinjiang Medical University, Urumqi 830011, China; 5 Xinjiang Key Laboratory of Clinical Drug Research, Urumqi 830011, China; 6 Cardiovascular Center, Affiliated Traditional Chinese Medicine Hospital of Xinjiang Medical University, Urumqi 830099, China; 7 School of Traditional Chinese Medicine, Capital Medical University, Beijing 100069, China

**Keywords:** electrochemical sensor, Au–Pt nanostructure, hierarchical bimetallic film, salvianolic acid B, *Salvia miltiorrhiza*

## Abstract

Salvianolic acid B is an important bioactive biomarker in traditional Chinese medicines (TCMs). Current methods for its quantitation are time- and cost-consuming; a rapid and reliable method is urgently needed. In this work, we have developed a simple and sensitive electrochemical (EC) sensor on screen-printed carbon electrode (SPCE) that was modified with three-dimensional hierarchical Au–Pt nanostructured film. This highly conductive structure facilitates direct electron transfer when the sample solution containing salvianolic acid B is dropped, thereby amplifying its generated current response. Under optimized conditions, the fabricated label-free EC sensor exhibited a wide linear range (4–500 μg/mL) with excellent conductivity and a detection limit as low as 3.76 μg/mL for salvianolic acid B, as well as high reproducibility and outstanding stability. Furthermore, the practical applicability was validated by satisfactory spiked recovery rates (97.5–101.4%) in complex *Salvia miltiorrhiza* matrices. These findings indicated that the Au–Pt nanostructure-modified SPCE could achieve reliable and stable quantitation of salvianolic acid B. The developed EC sensor provided an economical, efficient, user-friendly, and reliable on-site strategy for accurate determination of salvianolic acid B and other components in more TCMs.

## 1. Introduction

As a treasure of Chinese civilization, the importance of traditional Chinese medicine (TCM) has garnered increasing global recognition and gradually been accepted around the world. For instance, the successful approval of the Investigational New Drug (IND) application for Compound Danshen Dripping Pills (a patented TCM product derived from *Salvia miltiorrhiza*) by the U.S. Food and Drug Administration (FDA) in December 1997 underscores the critical need for rigorous quality control of active ingredients in TCM. Salvianolic acid B is a major water-soluble polyphenolic compound extracted from *Salvia miltiorrhiza*, and is present in relatively high abundance [1]. Numerous studies have demonstrated that Salvianolic acid B possesses a broad spectrum of biological activities, including anti-inflammatory [2,3], antioxidant [4,5], and anti-apoptosis [6] activities, etc. It can alleviate myocardial injury caused by ischemia [7,8] and hypoxia [9,10], inhibit vasoconstriction [11,12], and combat atherosclerosis [13,14], thereby playing a pivotal role in treating various clinical diseases [15,16].

The content of salvianolic acid B in *Salvia miltiorrhiza* is significantly influenced by multiple factors, such as geographic origin, variety, cultivation practices, growth stage, and post-harvest processing. Consequently, sensitive and rapid analytical methods for its determination are essential for quality control of crude drugs, industrial production of compound formulations, and pharmacological mechanism studies, which also facilitate the evaluation and authentication of medicinal materials. Currently, high-performance liquid chromatography [17,18,19], infrared spectroscopy [20,21], capillary electrophoresis [22,23], and thin-layer chromatography [24] are commonly used for the determination of the content of salvianolic acid B. Although exhibiting some advantages, they suffer from large and heavy instruments, tedious sample preparation, high cost and complex operation.

Electrochemical (EC) sensors represent a modern analytical technique based on the interaction between the analyte and a specifically functionalized modified interface, which generates an electrical signal proportional to the target concentration [25]. By recording the electrical signal, the qualitative or quantitative analysis of targets can be achieved [26]. When redox reactions occur, electrical energy is either released or consumed [27], enabling sensitive detection of the target. EC sensors offer distinct advantages, including device miniaturization, rapid response, high sensitivity, low cost, and ease of automation [28], making them a highly active research area. In fabricating an EC platform, the design of the sensing interface, particularly the substrate material, is a key factor in enhancing sensitivity, increasing active sites, and improving the overall detection performance. The hydrogen bubble dynamic template (HBDT) [29] method has emerged as a powerful and facile strategy for constructing hierarchical metallic nanostructures on electrode surfaces. This approach generates a large number of hydrogen bubbles as dynamic templates via water electrolysis under extremely high cathodic overpotential, directing metal ion reduction at the gas–liquid–solid three-phase interface and enabling one-step electrodeposition of porous films without the need for complex equipment or post-processing steps [30]. This versatile strategy can be applied to various metal combinations, among which bimetallic systems are of particular interest. Notably, gold–platinum (Au–Pt) nanostructures are especially attractive for electrochemical sensing applications due to their outstanding electrical conductivity, excellent electrocatalytic activity toward redox reactions, and synergistic effects arising from the combination of the two noble metals. The incorporation of Pt enhances the catalytic efficiency, while Au provides a stable conductive scaffold and serves as an ideal platform for the immobilization of functional biomolecules. Moreover, the three-dimensional hierarchical architecture further increases the electrochemically active surface area and provides abundant active sites, collectively contributing to significantly amplified current responses and improved detection sensitivity.

**Scheme 1:**
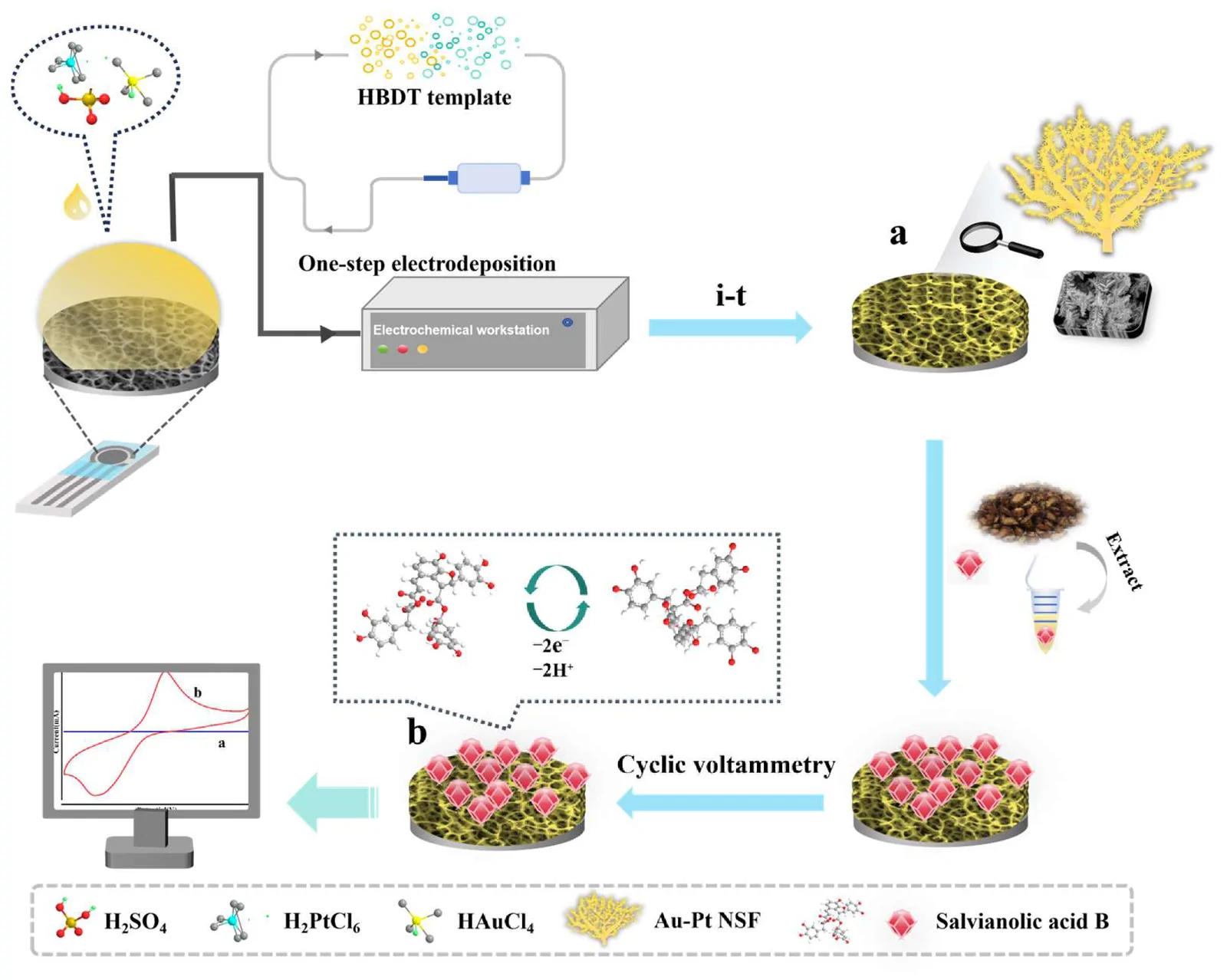
Fabrication of the hierarchical Au–Pt NSF-based EC sensor for salvianolic acid B detection.

In this study, we successfully synthesized a hierarchical Au–Pt nanostructured film (Au–Pt NSF) directly onto the surface of screen-printed carbon electrodes (SPCEs) via a simple one-step HBDT electrodeposition method. The fabricated Au–Pt NSF/SPCE was then employed as a label-free electrochemical sensing platform for the highly sensitive determination of salvianolic acid B (Scheme 1). Although the present platform did not incorporate a conventional biorecognition element (e.g., enzyme, antibody, or aptamer), the hierarchical Au–Pt nanostructured film functioned as an artificial recognition and catalytic interface for the selective detection of the bioactive component salvianolic acid B. Consequently, this nanomaterial-driven electrochemical sensor aligned with the biosensing scope by providing a rapid, label-free, and field-deployable strategy for pharmaceutical quality control and medicinal analysis. The electrochemical redox behavior and associated kinetic parameters of salvianolic acid B on the modified electrode were systematically investigated. Under optimized conditions, the developed sensor exhibited a wide linear range (4–500 μg/mL) with a low detection limit of 3.76 μg/mL, along with excellent reproducibility, repeatability, stability, and specificity. Furthermore, the practical applicability of the sensor was validated by successful quantification of salvianolic acid B in authentic *Salvia miltiorrhiza* samples, with recoveries ranging from 97.5% to 101.4%, and the results were highly consistent with those obtained by high-performance liquid chromatography (HPLC), demonstrating its reliability and accuracy for quality control applications in traditional Chinese medicines.

## 2. Materials and Methods

### 2.1. Reagents and Instrument

All reagents used were of analytical grade, and ultrapure water (resistivity > 18 MΩ·cm) was used throughout the experiments. Salvianolic acid B standard was purchased from Hunan Qingchun Technology Co., Ltd. (Changsha, China). Chloroauric acid hydrate (HAuCl_4_·4H_2_O) and chloroplatinic acid hexahydrate (H_2_PtCl_6_·6H_2_O) were obtained from Sinopharm Chemical Reagent Co., Ltd. (Shanghai, China). Potassium ferrocyanide (K_3_[Fe(CN)_6_]), potassium chloride, hydrochloric acid, acetonitrile, phosphoric acid, sulfuric acid, methanol, ethanol, and 0.1 M phosphate buffer solution (PBS, pH 7.2–7.4) were obtained from McLean Biochemical Co., Ltd. (Shanghai, China). Sodium hydroxide was bought from Chengdu Huayi Pharmaceutical Accessories Manufacturing Co., Ltd. (Chengdu, China).

All EC measurements were performed using a CHI 660E electrochemical workstation (Shanghai Chenhua Instrument Co., Ltd.) (Shanghai, China). Screen-printed carbon electrodes (SPCEs) incorporating a three-electrode system comprising a carbon working electrode (4 mm in diameter), an Ag/AgCl reference electrode, and a counter electrode were obtained from Weihai Putian Technology Co., Ltd. (Weihai, China) All cyclic voltammetry (CV) measurements in this experiment were conducted in a 0.1 M KCl solution containing 5 mM [Fe(CN)_6_]^3−/4−^, with an initial potential of −0.9 V and a termination potential of +0.9 V, a sampling interval of 0.001 V, and a scan rate of 100 mV/s in the positive direction. Surface morphology characterization was performed using a field-emission scanning electron microscopy (FE-SEM, ZEISS Sigma 360, Carl Zeiss, Oberkochen, Germany). Mass measurements were carried out on an electronic analytical balance (Sartorius ME215S, Sartorius AG, Göttingen, Germany).

### 2.2. Synthesis of Au–Pt NSF

The Au–Pt NSF was synthesized on a SPCE surface using the HBDT method based on the protocol described by Gao et al. with minor modification [31]. Briefly, the SPCE was exposed to 0.5 M H_2_SO_4_ and continuously scanned at a rate of 0.10 V/s within a voltage range of −0.2 to 0.12 V until a stable cyclic voltammetry (CV) curve was obtained. Subsequently, the Au–Pt NSF was prepared by potentiostatic electrodeposition (Amperometric i–t curve) by immersing the SPCE in a mixture of 6 mM HAuCl_4_·4H_2_O, 0.16 mM H_2_PtCl_6_·6H_2_O, and 2 M sulfuric acid at a constant potential of −4 V for 300 s.

In contrast to the rime-like Au–Pt film coated with polyaniline-based molecularly imprinted polymer used in [31], the current work constructed a label-free and polymer-free hierarchical Au–Pt NSF that served as a direct electrocatalytic interface for salvianolic acid B. In this configuration, the Pt component provided catalytically active sites for the electrochemical oxidation of the catechol moiety, while the Au skeleton ensured a highly conductive and stable scaffold for efficient interfacial electron transfer, thereby synergistically amplifying the redox peak currents of salvianolic acid B. Accordingly, the deposition parameters were systematically re-optimized for this new material configuration (Section 3.3). Notably, the present study provided a comprehensive kinetic analysis of the adsorption-controlled quasi-reversible redox process of salvianolic acid B on the hierarchical bimetallic surface (Section 3.2).

### 2.3. Sample Preparation

A stock solution of salvianolic acid B was prepared by dissolving the standard compound in 80% methanol, and working standards of various concentrations were obtained by serial dilution.

Three batches of *Salvia miltiorrhiza* decoction pieces were purchased from different manufacturers with sample batches Nos. 250301, 260502, and 230902. For real-sample analysis, 5 g of *Salvia miltiorrhiza* decoction pieces from three different manufacturers were accurately weighed and placed into a 250 mL glass flask. Then, 50% methanol was added to a total volume of 100 mL, and the mixture was heated under reflux in a water bath for 0.5 h, followed by cooling to room temperature. The extract was filtered through gauze, and 1 mL of the filtrate was diluted to 10 mL with 50% methanol in a volumetric flask to obtain the sample solution, which was stored at 4 °C and analyzed within 3 days of preparation. Prior to analysis, 1 mL of this solution was centrifuged at 8000 rpm for 10 min, and the supernatant was collected for the following testing.

To ensure statistical reliability, all electrochemical measurements were carried out in triplicate (*n* = 3), with each run using a freshly prepared modified electrode and freshly prepared sample solutions. The results are expressed as mean ± standard deviation (SD), and all error bars in the figures represent the SD of three independent experiments.

## 3. Results

### 3.1. Characterization of Thin Au–Pt NSF

#### 3.1.1. Material Characterization and Testing Principles

**Figure 1:**
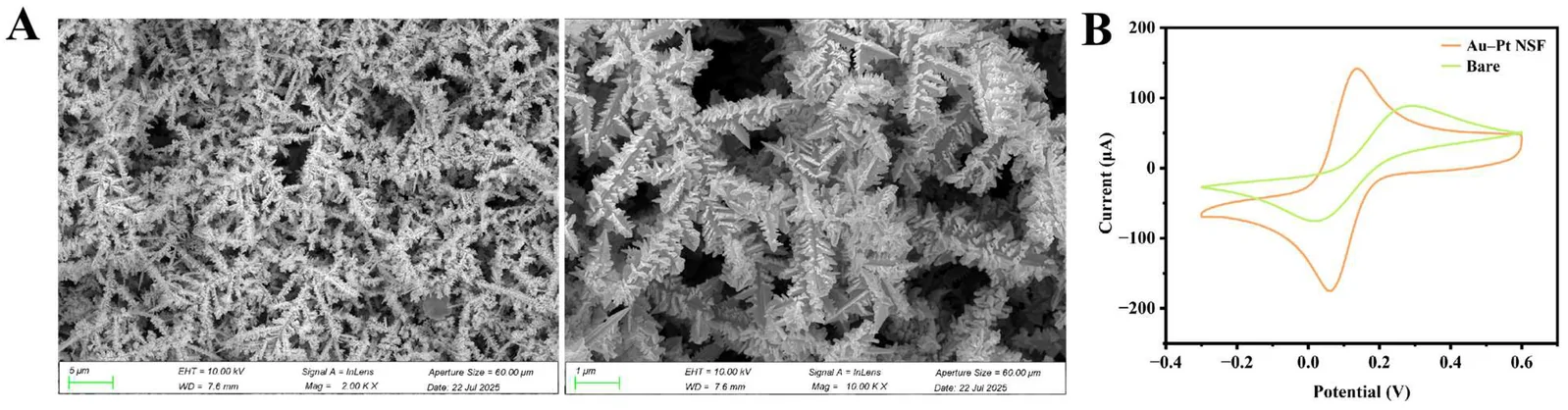
(**A**) SEM image displays the surface features of Au–Pt NSF; (**B**) CV of bare and Au–Pt NSF.

As shown in Figure 1A, the SEM image reveals the surface morphology of the Au–Pt NSF. The electrodeposited hierarchical Au–Pt NSF formed on the electrode surface exhibits dendritic frameworks and nanoscale protrusions, indicating high surface roughness and a three-dimensional (3D) hierarchical architecture. This structure significantly increases the specific surface area and provides abundant active sites for EC reactions. Figure 1B showed the comparison of the CV responses between the Au–Pt NSF-modified electrode and the bare carbon electrode, further confirming the enhanced EC performance.

In this study, the Au–Pt NSF was employed to improve both EC sensitivity and detection capability. When a solution of salvianolic acid B is dropped onto the modified electrode surface and a voltage is applied, the catechol moiety of salvianolic acid B undergoes electron transfer. This process involves the sequential loss of one electron and one proton from the catechol group to form a semiquinone radical, which then loses another electron and proton to yield a stable quinone product. This redox process gives rise to characteristic oxidation and reduction peaks. As the concentration of salvianolic acid B increases, more electrons are transferred, resulting in a higher peak current.

#### 3.1.2. Electrode Surface Active Area

The electroactive surface area (*A*) of the bare and Au–Pt NSF-modified SPCE was determined by cyclic voltammetry using 5 mM [Fe(CN)_6_]^3−^/^4−^ in 0.1 M KCl as the redox probe (Figure 1B). According to the Randles-Ševčík equation for a reversible process at 25 °C:

(1) $$\begin{array}{l} A=\frac{I_{P}}{\left(2.69\times 10^{5}\times n^{\frac{3}{2}}\times D^{\frac{1}{2}}\times v^{\frac{1}{2}}\times C\right)} \end{array}$$

where *I*_p_ represents the peak current (1.65 × 10^−4^ A), *A* is the electroactive area (cm^2^), *D* is the diffusion coefficient (6.7 ± 0.02 × 10^−6^ cm^2^·s^−1^), *n* is the number of electrons transferred (*n* = 1 for [Fe(CN)_6_]^3−^/^4−^), *v* is the scan rate (0.05 V/s), and *C* is the concentration of probe molecules (5 × 10^−6^ mol·cm^−3^ [Fe (CN)_6_]^3−/4−^). Substituting these values into equation (1), *A* was calculated to be 0.212 cm^2^ for the Au–Pt NSF/SPCE, which is approximately 2.35-fold larger than that of the bare SPCE (0.0903 cm^2^). This significant increase confirms that the three-dimensional hierarchical Au–Pt nanostructure enhances the electrode surface roughness and provides abundant accessible active sites for subsequent electrochemical reactions.

### 3.2. Electrooxidation Mechanism of Target Salvianolic Acid B

#### 3.2.1. Redox Peak of Salvianolic Acid B

**Figure 2:**
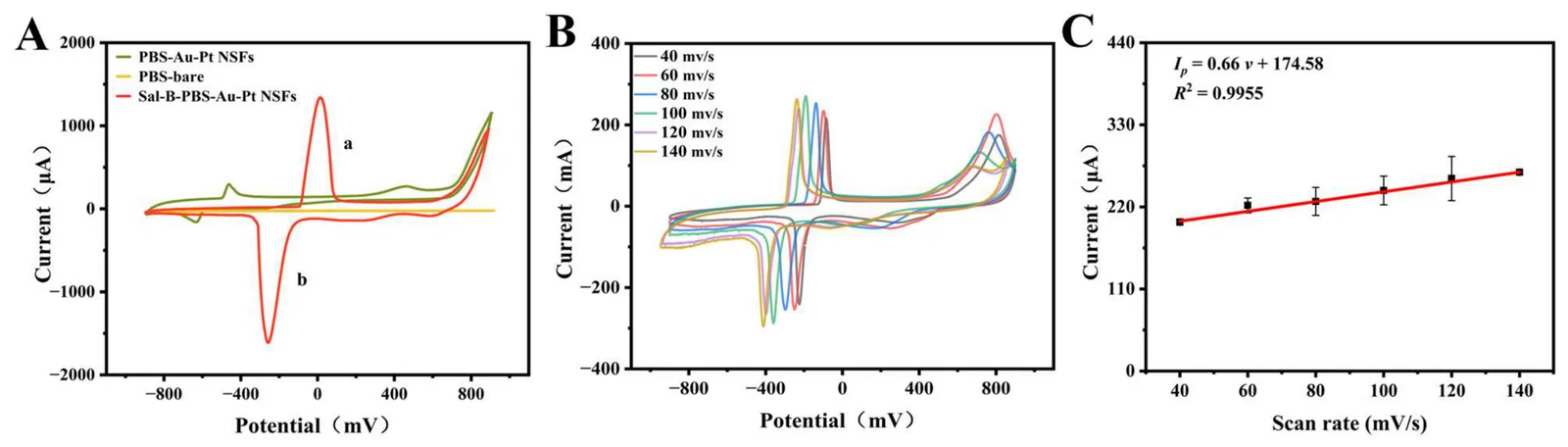
(**A**) A pair of distinct oxidation-reduction peaks of salvianolic acid B detected on the surface of Au–Pt NSF/SPCE; a:oxidation peak, b:reduction peak; (**B**) CV of salvianolic acid B with different scan rate; (**C**) the scan rate and peak current show a good linear relationship equation.

Using CHI 660E electrochemical workstation, the EC behavior of salvianolic acid B on the Au–Pt NSF-modified SPCE surface was investigated through CV determination in the potential range of −0.9 to +0.9 V (Figure 2A). Salvianolic acid B solution in PBS buffer solution was found to exhibit a well-defined pair of redox peaks.

At a scanning rate of 100 mV/s, the peak potential separation (Δ*E* = *E*_pa_ − *E*_pc_) was 280 mV, which deviates from the theoretical value of 59/n mV for a reversible system. At the same time, the current ratio of the anodic-to-cathodic peak is *I_pa_*/*I_pc_* = 1.206 mA/1.490 mA = 0.8094 < 1, and the peaks are asymmetric, indicating that the redox reaction of salvianolic acid B on the Au–Pt NSF/SPCE exhibits a quasi-reversible process.

#### 3.2.2. Effect of Scan Rate on Redox Peaks

To investigate the electrocatalytic redox kinetics parameters of salvianolic acid B on the fabricated EC sensor, CV measurements were performed in a PBS solution containing salvianolic acid B (pH 7.0) at a potential range from −0.9 V to +0.9 V, using a freshly prepared Au–Pt NSF/SPCE for each scan rate within the range of 40–140 mV/s. The potential window from −0.9 V to +0.9 V was chosen to fully encompass the redox peaks of salvianolic acid B (as shown in Figure 2A, a:oxidation peak, b:reduction peak) while avoiding hydrogen/oxygen evolution and background current interference from electrolyte decomposition. This protocol was adopted to prevent cumulative surface fouling or alteration of the adsorbed layer during successive scans, thereby ensuring that each current response reflects the intrinsic scan-rate dependence of the adsorption-controlled redox process. The relationship curve between peak current *Ip* and scan rate is shown in Figure 2B. With an increased scan rate, both the anodic and cathodic peak currents of salvianolic acid B on the Au–Pt NSF/SPCE increase significantly, and the redox peak potentials shift negatively. A good linear relationship was observed between the peak current (*I_p_*) and the scan rate (*v*) with the regression equation *I_p_* = 0.66 *v* + 174.58 (*R*^2^ = 0.9955) (Figure 2C). This result indicates that the electrocatalytic redox kinetics of salvianolic acid B under Au–Pt NSF/SPCE conditions are controlled by dynamic adsorption. It is noteworthy that the redox peak potentials shift only slightly negatively with an increasing scan rate (ΔE ≈ 10–20 mV across 40–140 mV/s). This modest potential dependence is characteristic of a surface-confined, adsorption-controlled redox process, where the peak position is primarily determined by the interfacial electron-transfer kinetics of the adsorbed species rather than by diffusion-layer dynamics.

#### 3.2.3. Quantitative Evaluation of Adsorption-Controlled Kinetics

As shown in Figure 2A, the CV measurement of salvianolic acid B on the Au–Pt NSF/SPCE within the potential window from −0.9 V to +0.9 V exhibited only a single pair of redox peaks, indicating that only one catechol unit participated in the reversible redox reaction under the present conditions. The other units in the salvianolic acid B molecule are not electrochemically accessible within this potential window. To gain deeper insight into the adsorption-controlled process, the surface coverage (Γ) of salvianolic acid B on the Au–Pt NSF/SPCE was quantitatively estimated from the slope of the *Ip* vs. *v* plot (Figure 2C). For an adsorption-controlled redox reaction, the peak current follows the equation:

(2) $$Ip=\frac{n^{2}F^{2}}{4RT}Av\Gamma$$

where *n* = 2 (two-electron/two-proton transfer from a single catechol moiety to the corresponding quinone), *F* is the Faraday constant (96,485 C·mol^−1^), *R* is the gas constant (8.314 J·mol^−1^·K^−1^), T is the absolute temperature (298 K), A is the electroactive area (0.212 cm^2^), and *v* is the scan rate. The slope of *Ip* versus *v* obtained from Figure 2C is 0.66 μA/(mV·s^−1^), that is, 6.6 × 10^−4^ A·V^−1^·s. Substituting these values into Equation (2) gives: Γ = 8.28 × 10^−10^ mol·cm^−2^. This relatively high surface coverage indicates that the hierarchical Au–Pt nanostructure effectively enriches salvianolic acid B molecules on the electrode surface through strong adsorption interactions, which is fully consistent with the adsorption-controlled kinetic behavior.

Furthermore, the apparent heterogeneous electron transfer rate constant (*k_s_*) was estimated using the Laviron theory for quasi-reversible systems [32]. Based on the peak-to-peak separation (*ΔEp* = *Epa* − *Epc* = 0.280 V) at a scan rate of 100 mV·s^−1^, with *Epa* = 0.019 V and *Epc* = −0.261 V (extracted from Figure 2A), and assuming a symmetric charge transfer coefficient (α = 0.5), the *k_s_* value was calculated to be 1.66 × 10^−2^ s^−1^ according to the Laviron equation. This low *ks* value further confirms the quasi-reversible nature of the salvianolic acid B redox process on the Au–Pt NSF/SPCE. The combination of a high surface coverage and a moderate electron transfer rate constant demonstrates that the bimetallic Au–Pt nanostructure not only provides sufficient active sites for analyte but also maintains favorable interfacial electron transfer kinetics, synergistically contributing to the enhanced sensing performance.

### 3.3. Optimization of Experimental Conditions

Some key parameters including deposition time, HAuCl_4_·4H_2_O concentration, and solution pH were systematically optimized to maximize the sensing performance.

#### 3.3.1. Deposition Time

**Figure 3:**
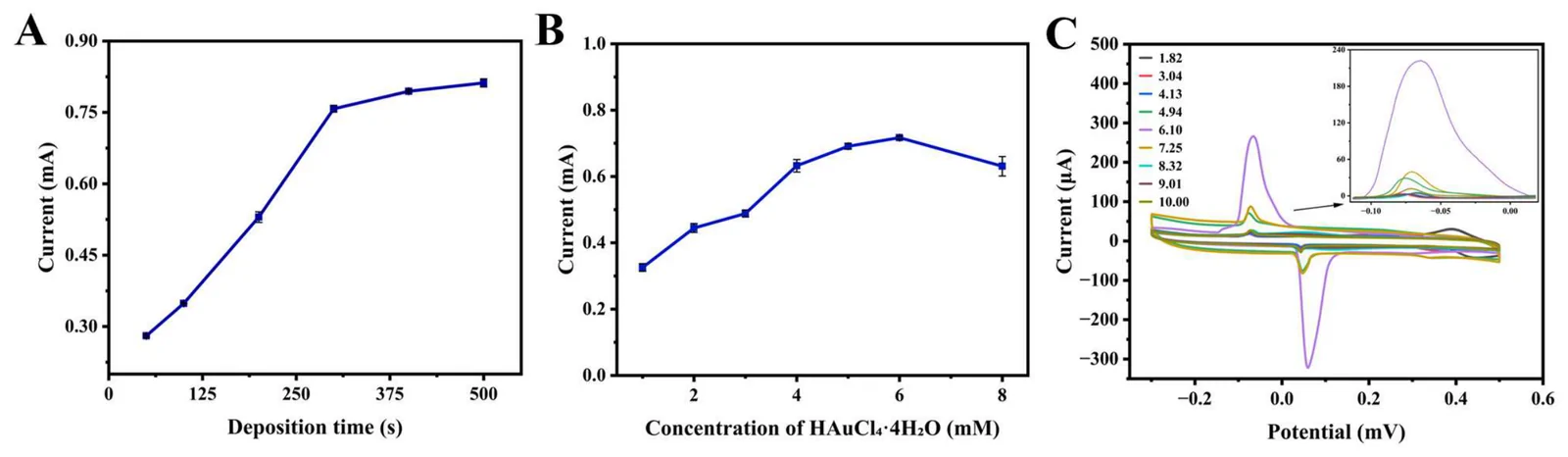
Optimization of (**A**) deposition time of Au–Pt NSF, (**B**) concentration of HAuCl_4_·4H_2_O, and (**C**) solution pH.

The deposition time directly influences the thickness and morphology of the Au–Pt NSF. Short deposition time can lead to thin alloy film, unclear improvement in conductivity, and failure to meet low concentration detection requirements. However, too long a deposition time will induce too many cavities during the deposition process, hindering the diffusion of electrolytes and electron transfer. Thus, Au–Pt deposition solution at the same concentration was dropped onto the SPCE surface and performed for durations of 50, 100, 200, 300, 400, and 500 s, respectively. The deposition effect was tested in a 5 mM [Fe(CN)_6_]^3−/4−^ solution. The results showed that as the deposition time increased, the current intensity increased (Figure 3A). At 300 s of deposition, the Au–Pt NSF exhibited the strongest conductivity, and when the time exceeded 300 s, the increase in current intensity leveled off with time. Therefore, 300 s was selected as the optimal deposition time.

#### 3.3.2. HAuCl_4_·4H_2_O Concentration

The SPCE used in this work exhibits relatively poor conductivity as a three-electrode system. The ratio of HAuCl_4_·4H_2_O to H_2_PtCl_6_·6H_2_O can significantly affect the electrode conductivity, so it is necessary to investigate the concentration of HAuCl_4_·4H_2_O to optimize the ratio. The mixed Au–Pt solution with different concentrations of HAuCl_4_·4H_2_O was deposited onto the SPCE surface for 300 s. Using a 5 mM [Fe(CN)_6_]^3−/4−^ solution, the optimal deposition effect at a certain concentration was detected by differential pulse voltammetry (DPV), as shown in Figure 3B. The results indicate that the current response significantly increases with the increase of HAuCl_4_·4H_2_O concentration in the range of 1 mM Au and 0.16 mM Pt to 6 mM Au and 0.16 mM Pt. When the HAuCl_4_·4H_2_O concentration is greater than 6 mM, the current intensity decreases, which may be due to the high concentration of HAuCl_4_·4H_2_O disrupting the synergistic effect between gold and platinum and reducing the electron transfer efficiency. Thus, the final optimal deposition solution ratio was determined to be 6 mM HAuCl_4_·4H_2_O and 0.16 mM H_2_PtCl_6_·6H_2_O.

#### 3.3.3. Solution pH

The detection solution pH plays a crucial role affecting the EC response characteristics, detection sensitivity, and chemical stability of the target analyte. Solution pH regulates the ionization state of phenolic hydroxyl groups and the electron transfer rate on the electrode surface, determining the magnitude of the oxidation peak current and detection sensitivity. Additionally, solution pH influences the chemical stability of salvianolic acid B in the analytical solution, directly affecting the accuracy of the measurement results. Therefore, it is necessary to investigate the pH conditions when using electrochemical methods to detect the concentration of salvianolic acid B. Figure 3C shows that when the pH is set at 6, the current conductivity is significantly higher than those under other acidic and alkaline pH conditions. From pH 2 to 6, the current value gradually increases and the potential moves forward; beyond pH 6, both the current and potential decreased with increasing alkalinity. Therefore, pH 6.0 was selected as optimal for salvianolic acid B detection.

### 3.4. Method Validation for Salvianolic Acid B Detection

#### 3.4.1. Linearity and Limit of Detection

**Figure 4:**
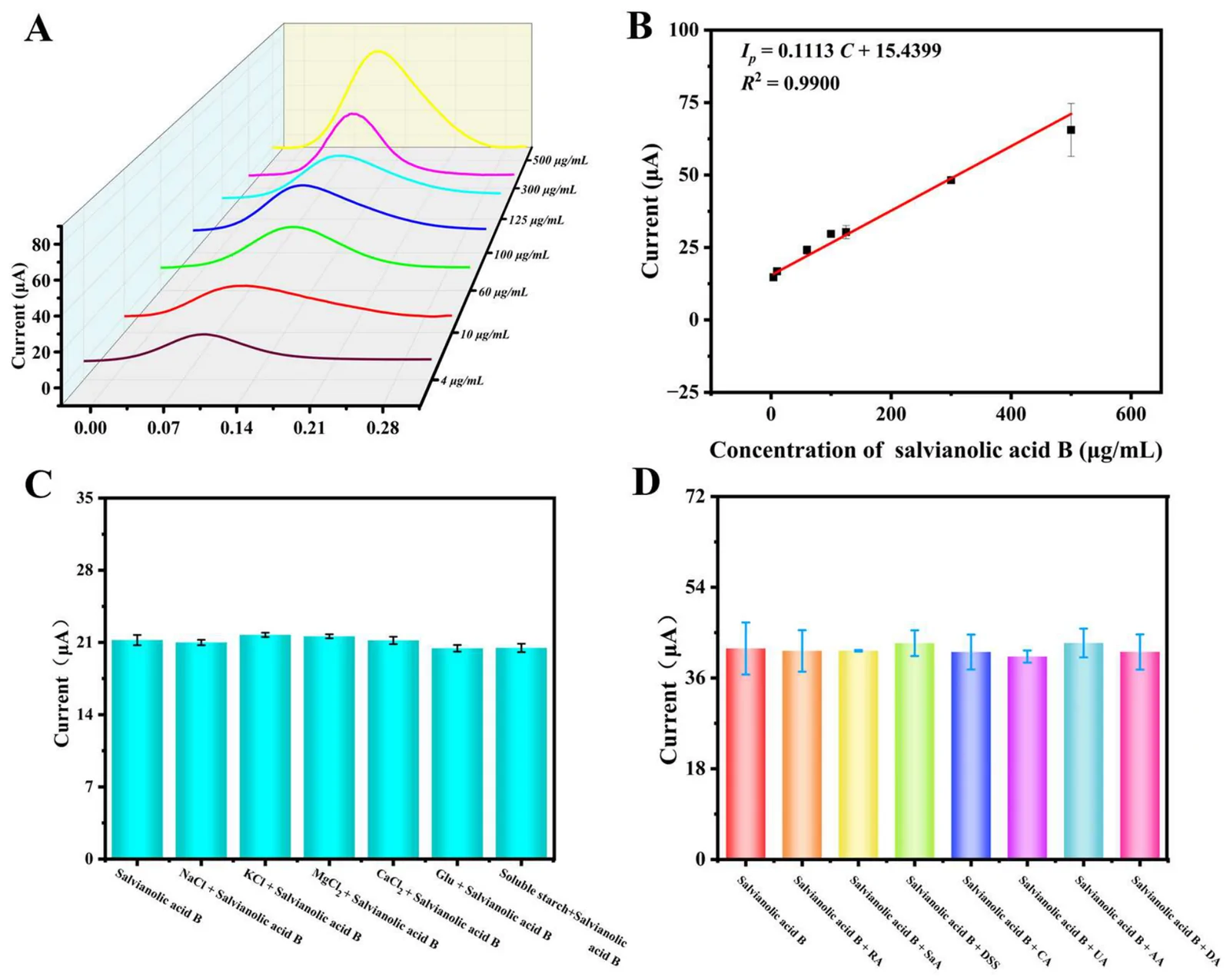
(**A**) DPV curves after rebinding of salvianolic acid B standard solutions across a concentration range of 4 μg/mL to 500 μg/mL (4, 10, 60, 100, 125, 300, 500 μg/m); (**B**) the corresponding calibration curve of ΔI versus the concentration of salvianolic acid B; (**C**) specificity; (**D**) anti-interference performance.

The standard solution of salvianolic acid B was dissolved in PBS solution to prepare test solutions with concentrations ranging from 4 to 500 μg/mL, and the DPV curves of a series of different concentrations of salvianolic acid B standard solutions were measured in Figure 4A. As shown in Figure 4B, there is a good linear relationship between the oxidation peak current and concentration of salvianolic acid B. The regression equation between the oxidation peak current of salvianolic acid B and its concentration is *I_p_* = 0.1113 *C* + 15.4399 (*R*^2^ = 0.9900) in a linear range of 4–500 μg/mL. The limit of detection (LOD) was calculated to be 3.76 μg/mL based on the formula LOD = 3σ/S, where σ is the standard deviation of the blank response (*n* = 3) and S is the slope of the calibration curve, demonstrating the excellent sensitivity of the Au–Pt NSF/SPCE-based EC sensor.

#### 3.4.2. Selectivity

Selectivity is a crucial factor affecting the performance of the proposed sensor. In order to explore the impact of interfering substances on the detection of salvianolic acid B, especially in the quality control of TCMs, common interfering substances potentially presented in *Salvia miltiorrhiza* extracts were investigated via the DPV method using the sensor (Figure 4C,D). Firstly, NaCl, KCl, MgCl_2_, CaCl_2_, glucose, and starch were determined (Figure 4C). The specific concentrations of the substances were set as 800 μM for inorganic ions and glucose, and 267 μg/mL for starch, so as to match or exceed the expected levels of these species in the diluted *Salvia miltiorrhiza* extract prepared according to Section 2.3, thereby ensuring that the anti-interference test reflects realistic matrix conditions. During the detection process, it could be seen that the added interfering ions did not have a significant effect on the determination of salvianolic acid B by using the Au–Pt NSF/SPCE-based EC sensing platform. The oxidation peak current of salvianolic acid B showed excellent stability with an RSD of 2.22%.

To further evaluate the selectivity of the proposed sensor for detecting salvianolic acid B, the effects of common coexisting compound components in *Salvia miltiorrhiza* and potential interfering substances with similar groups or oxidant potentials were studied. As shown in Figure 4D, upon separately adding 240 μg/mL of rosmarinic acid (RA), salvianolic acid A (SaA), danshensu (DSS), ascorbic acid (AA), and dopamine (DA), and 50 μg/mL (affected by solubility in water) caffeic acid (CA) and uric acid (UA) to 240 μg/mL of salvianolic acid B, the current intensity of each group remained similar to the individual detection of salvianolic acid B (RSD = 2.195%), without significant changes observed. The results indicated that potential interfering substances did not significantly interfere with the salvianolic acid B. The established Au–Pt NSF sensor showed good anti-interference ability and selectivity, and can be used for accurate detection of salvianolic acid B in complex matrix. The observed selectivity was presumably attributable to the specific adsorption affinity of salvianolic acid B toward the hierarchical bimetallic surface, as well as the optimized detection potential and pH conditions, which collectively minimized the electrochemical interference from co-existing phenolic analogues.

#### 3.4.3. Accuracy

The practical applicability and accuracy of the proposed Au–Pt NSF/SPCE sensor were further evaluated by standard addition recovery experiments. Five different concentrations of salvianolic acid B (20, 80, 145, 180, and 350 μg/mL) were spiked into three batches of *Salvia miltiorrhiza* water extracts, and each spiked sample was measured in triplicate. As summarized in Table 1, the recoveries ranged from 97.5% to 101.4%, with relative standard deviations (RSDs) between 0.32% and 4.88% (all < 5.0%, *n* = 3). It is worth noting that the concentration of the detection results is very consistent with the theoretical spiked value, indicating that the sensor does not have significant matrix interference. These results confirm that the Au–Pt NSF/SPCE method offers reliable and accurate quantification of salvianolic acid B in complex herbal matrices, with performance comparable to established analytical techniques.

#### 3.4.4. Reproducibility, Repeatability, and Stability

**Figure 5:**
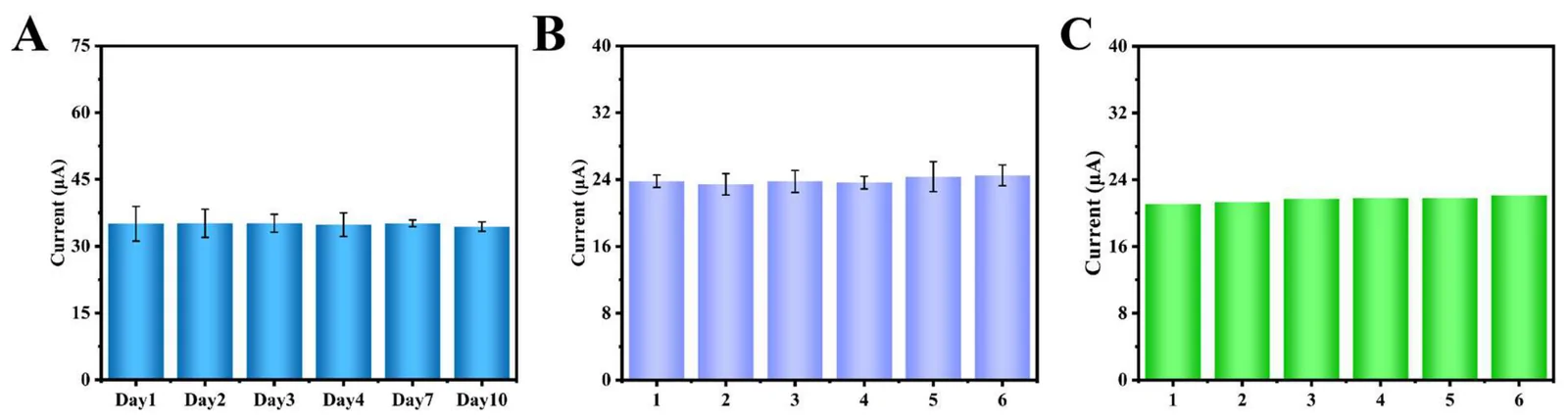
(**A**) stability, (**B**) reproducibility, and (**C**) repeatability of Au–Pt NSF/SPCE.

Six independently prepared SPCE electrodes were tested under the optimal conditions to evaluate the reproducibility of these electrodes using the same standard solution of salvianolic acid B. Figure 5B exhibited small RSD of the current responses of 1.58%, indicating good reproducibility. In addition, a standard solution of salvianolic acid B was continuously measured using the same modified electrode to evaluate the repeatability of Au–Pt NSF. The results showed that six consecutive measurements with the same electrode yielded an RSD of 1.50% (Figure 5C), which demonstrated excellent repeatability of the Au–Pt NSF modified SPCE electrode.

The prepared Au–Pt NSF/SPCE was placed in a 10 mL centrifuge tube and stored at room temperature without any special treatment. The prepared sensors were measured for different storage days, and 175 μg/mL of salvianolic acid B stock solution was detected. On the 10th day, the peak oxidation-reduction current was 98.18% of its initial value, with an RSD of 0.78% (Figure 5A), confirming the good stability of the developed EC sensor.

### 3.5. Real-Sample Analysis

#### 3.5.1. Detection of Salvianolic Acid B in Real *Salvia miltiorrhiza* Samples

To validate the accuracy of the developed Au–Pt NSF/SPCE EC sensor, the contents of salvianolic acid B in three batches of *Salvia miltiorrhiza* decoction pieces from different manufacturers were determined, and the results are shown in Table 2. The results obtained by the two methods were in good agreement, with relative deviations of −0.61%, −0.88%, and −0.48% for Sample 1, Sample 2, and Sample 3, respectively. The electrochemical method yielded mean concentrations of 181.24, 93.22, and 70.19 μg/mL for the three samples.

#### 3.5.2. HPLC Analysis of Salvianolic Acid B in *Salvia miltiorrhiza*

A Shimadzu LC–20A high-performance liquid chromatography was used to detect the content of salvianolic acid B in *Salvia miltiorrhiza* purchased in the market with different concentrations and ratios of acetonitrile/phosphoric acid and acetonitrile/formic acid being used as the mobile phases. After evaluating various mobile phase compositions, acetonitrile/0.1% phosphoric acid (22:78, *v*/*v*) was selected based on separation factor, tailing factor, and theoretical plate number. Chromatographic conditions were as follows: Elite C18 column (E3121502, 250 mm × 4.6 mm, 5.0 μm), PAD detector, flow rate of 1 mL/min, detection wavelength at 286 nm, column temperature at 30 °C, and injection volume of 10 μL. The separation of salvianolic acid B in the water extract of *Salvia miltiorrhiza* decoction pieces under these conditions is shown in Figure S1. The linear regression curve was presented in Figure S2. Salvianolic acid B was found to show an independent symmetrical peak at 27.2 min. The detection results were shown in Table 2, with contents of 182.35, 94.05, and 70.53 μg/mL, which were almost the same as those obtained from the newly developed EC sensor.

The small discrepancies between the two methods confirm that the developed Au–Pt NSF/SPCE-based EC sensor provides reliable quantification of salvianolic acid B in complex herbal matrices, with accuracy comparable to the established HPLC reference method. Notably, Sample 3 exhibited a higher RSD (6.20%) compared to Samples 1 and 2. This is primarily attributed to its lower salvianolic acid B concentration (70.19 µg/mL) and inherent batch-to-batch matrix variability among different manufacturers, which amplified relative uncertainty in replicate measurements. Nevertheless, the satisfactory recovery rates (Table 1) and the close agreement with HPLC validation (relative deviation: −0.48%) demonstrated that the sensor remained accurate and practical for real-sample analysis.

The analytical performance of the proposed DPV method was evaluated and compared with that of previously reported techniques for salvianolic acid B determination (Table 3). Currently, LC-MS/MS remains the predominant technique for this analyte due to its excellent sensitivity. However, its widespread application for routine quality control is considerably restricted by high instrumentation costs, labor-intensive operation, and substantial organic solvent consumption. Moreover, alternative rapid detection methods for salvianolic acid B are still scarce. In this context, the proposed DPV method achieves a detection limit of 3.76 µg/mL with a linear range of 4–500 µg/mL, which is entirely adequate for the quality control of herbal products given the relatively high abundance of this marker compound. More importantly, this method offers compelling practical advantages, including cost-effective instrumentation, streamlined sample preparation, minimal solvent usage, and strong potential for on-site or industrial process monitoring. These features collectively position the DPV method as a green, economical, and operationally convenient alternative to conventional chromatographic approaches. While several nanomaterial-modified GCE or CPE platforms have achieved lower LODs through pre-concentration or ultra-high surface-area materials, the present Au–Pt NSF/SPCE sensor distinguishes itself by balancing sensitivity with an exceptionally wide linear range and the practical advantages of disposable screen-printed electrodes, making it particularly suited for rapid, on-site screening of TCM matrices.

## 4. Conclusions

In this study, a hierarchical Au–Pt NSF was successfully synthesized and integrated with an SPCE to construct a highly sensitive EC sensor for sensitive detection of salvianolic acid B. The Au–Pt NSF possesses a large electrochemically active surface area, high porosity, and excellent electrical conductivity, which facilitate electron transfer and promote the redox process of salvianolic acid B. The screen-printed electrode offers the additional advantages of superior stability, portability, and cost-effectiveness. The combination of Au–Pt NSF and SPCE enables the rapid detection of salvianolic acid B. Several key parameters influencing the sensor performance were systematically optimized, and the results showed that the Au–Pt NSF/SPCE-based EC sensor achieved optimal detection performance at a deposition time of 300 s, a HAuCl_4_·4H_2_O concentration of 6 mM, a H_2_PtCl_6_·6H_2_O concentration of 0.16 mM, and a pH of 6.0. Electrochemical kinetic studies revealed that the redox reaction of salvianolic acid B on the Au–Pt NSF/SPCE is adsorption-controlled. The sensor exhibited a wide linear range (4–500 μg/mL), an ultralow limit of detection (3.76 μg/mL), and excellent reproducibility, stability, and selectivity. Its practical utility was validated by successfully quantifying salvianolic acid B in aqueous extracts of authentic *Salvia miltiorrhiza* slices, and the results obtained with this biosensor were consistent with those from HPLC analysis. In summary, considering its simple manufacturing, ease of operation, and powerful performance, this electrochemical sensor has great potential in the quality control of TCMs and can be extended to the detection of other bioactive compounds.

It should be noted that the real-sample validation was performed on three commercial batches of *Salvia miltiorrhiza* decoction pieces from different manufacturers. Although the results showed satisfactory recoveries (97.5–101.4%) and were highly consistent with the HPLC reference method (Table 2), the relatively limited number of batches represents a constraint on the generalizability of the method across the full diversity of TCM production. Future studies will expand the validation to a larger cohort of batches and geographic origins to further consolidate the reliability of this electrochemical sensor for industrial-scale quality control.

## Figures and Tables

**Table 1:** Recovery rate of salvianolic acid B in *Salvia miltiorrhiza* water extract (*n* = 3).

Sample	Added (μg/mL)	Found (μg/mL)	Recovery (%)	RSD (%, *n* = 3)
Sample 1	20	197.63 ± 2.7	97.7	1.37
	80	255.75 ± 3.35	97.5	1.31
	145	328.39 ± 2.63	100.3	0.80
	180	360.79 ± 7.50	99.5	2.08
	350	526.65 ± 11.67	98.9	2.21
Sample 2	20	115.31 ± 5.6	101.1	4.88
	80	174.99 ± 2.0	100.5	1.15
	145	238.11 ± 3.2	99.6	1.36
	180	275.36 ± 4.7	100.5	1.72
	350	442.15 ± 1.4	99.6	0.32
Sample 3	20	91.84 ± 2.5	101.4	2.74
	80	149.27 ± 5.1	99.2	3.42
	145	216.19 ± 0.8	100.3	0.38
	180	252.82 ± 5.2	100.9	2.04
	350	422.60 ± 9.5	100.5	2.25

Data are presented as mean ± SD (*n* = 3). RSD: relative standard deviation.

**Table 2:** Determination of salvianolic acid B in *Salvia miltiorrhiza* by the developed EC sensor and confirmed by the HPLC method.

Sample	EC Sensor (μg/mL)	SD (μg/mL)	RSD (*n* = 3)	HPLC (μg/mL)
Sample 1	181.24	1.13	0.62%	182.35
Sample 2	93.22	2.58	2.77%	94.05
Sample 3	70.19	4.35	6.20%	70.53

**Table 3:** Comparison of analytical performance of the developed EC sensor with other reported methods for salvianolic acid B detection.

Analyte	Technique	Range (µg/mL)	LOD (µg/mL)	Ref.
Salvianolic acid B	HSI SE-ResNet	13.420–2680	4.0260	[33]
	HPLC-DAD and NIR	21–1064	/	[34]
	UHPLC-Q-Orbitrap-HRMS	0.469–15.0	3.301 ×10^−2^	[35]
	1H-NMR	/	20	[36]
	UPLC-DAD	1.87–59.78	0.19	[37]
	HPLC-DAD-MS/MS	11.59–2318	3.23 × 10^−2^	[38]
	UHPLC	10.45–522.5	0.174	[39]
	DPV (Au@CeO_2_-Fe_3_O_4_/rGO/GCE)	0.72–718.6	0.027	[40]
	DPV (porous carbon/GCE)	0.005–1.0	0.0012	[41]
	DPV (bare GCE)	0.14–2.80	0.093	[42]
	DPV (Au–Pt NSF)	4–500	3.76	This work

HSI: Hyperspectral Imaging Analysis; SE-ResNet: Squeeze-and-Excitation Residual Networks; HPLC-DAD: high-performance liquid chromatography-diode array detector; NIR: near-infrared; UHPLC-Q-Orbitrap-HRMS: ultra-high performance liquid chromatography with Q Exactive hybrid quadrupole orbitrap high-resolution accurate mass spectrometry; 1H-NMR: Hydrogen-1 Nuclear Magnetic Resonance; DPV: differential pulse voltammetry.

## Data Availability

The original contributions presented in the study are included in the article; further inquiries can be directed to the corresponding author.

## Supplementary Materials

The following supporting information can be downloaded at https://www.mdpi.com/article/10.3390/bios16090508/s1, Figure S1: HPLC for water extract of Danshen decoction pieces; Figure S2: The corresponding calibration curve of the peak area of HPLC versus the concentration of salvianolic acid B.
