# Supplementary material for: Hierarchical Au–Pt Nanostructured Film-Enabled Electrochemical Sensor for Highly-Sensitive Determination of Salvianolic Acid B
Source: Biosensors (Basel). 2026 Sep 10;16(9):508. doi: 10.3390/bios16090508 (PMC13604074; doi:10.3390/bios16090508)
Supplement: Supplementary file 1 [file biosensors-16-00508-s001.zip › biosensors-4513092-supplementary.pdf]

Supplementary Materials

Hierarchical Au–Pt Nanostructured Film-Enabled Electrochemical Sensor for Highly-Sensitive Determination of Salvianolic Acid B

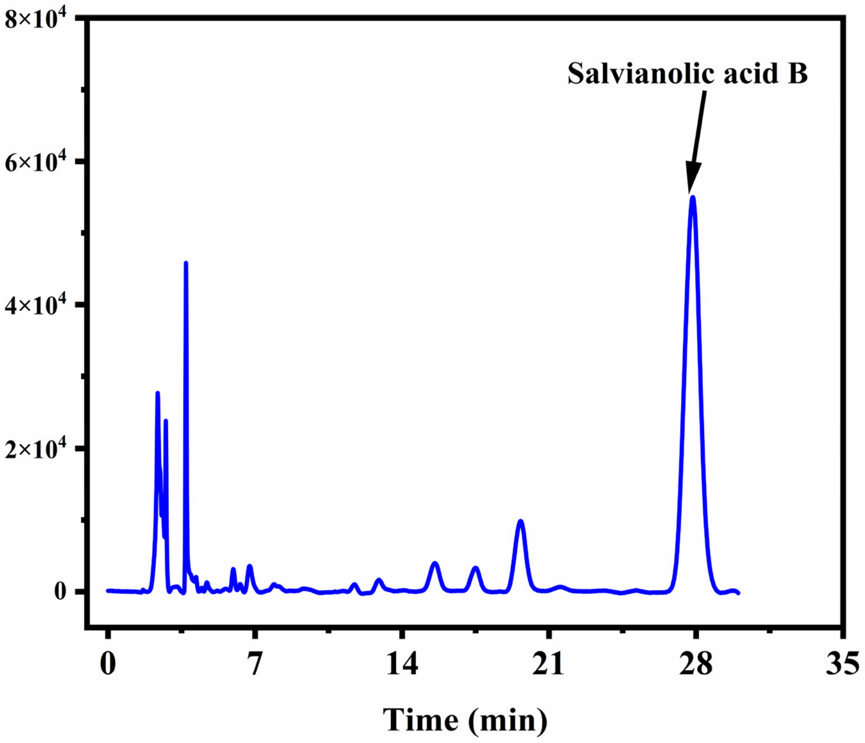

Figure S1. HPLC for water extract of Danshen decoction pieces.

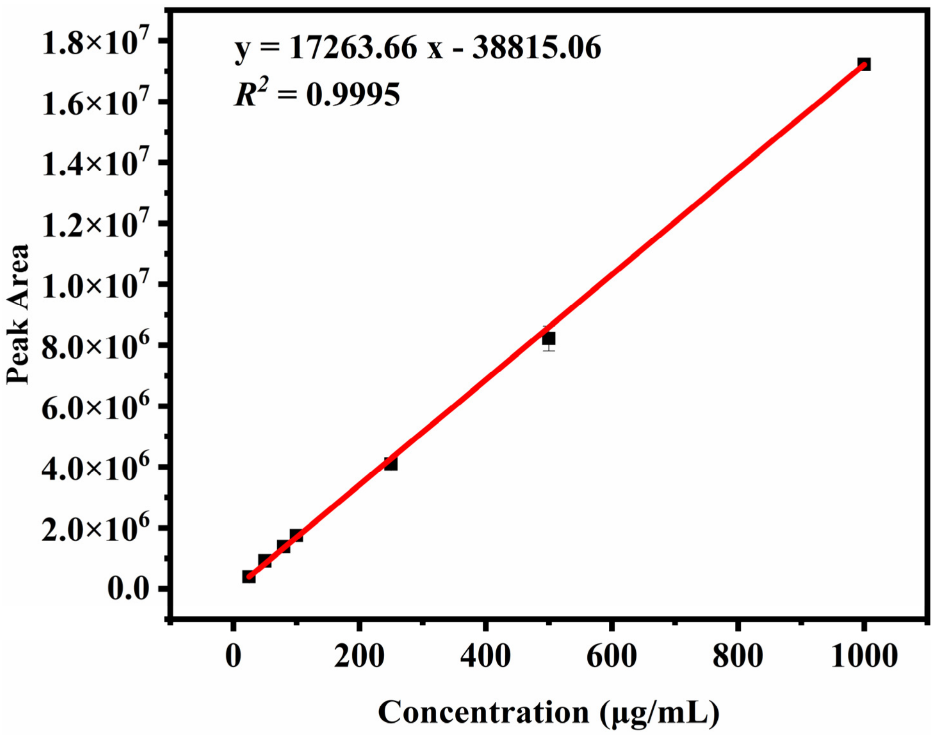

Figure S2. The corresponding calibration curve of peak area of HPLC versus the concentration of salvianolic acid.
